# Dynamic changes in depressive symptoms at the onset of military conflict in a neighboring country: a cross-sectional study

**DOI:** 10.3389/fpsyt.2025.1593088

**Published:** 2025-09-10

**Authors:** Artur Airapetian, Valentas Gruzauskas, Neringa Urbonaite, Benedikt Bachmetjev, Povilas Bernadickas, Laura Nedzinskiene, Rolandas Zablockis

**Affiliations:** ^1^ Vilnius University Faculty of Medicine, Vilnius University, Vilnius, Lithuania; ^2^ Institute of Computer Science, Vilnius University, Vilnius, Lithuania; ^3^ Institute of Data Science and Digital Technologies, Vilnius University, Vilnius, Lithuania; ^4^ Department of Anatomy, Histology and Anthropology, Institute of Biomedical Sciences, Faculty of Medicine, Vilnius University, Vilnius, Lithuania; ^5^ Clinic of Chest Diseases, Immunology and Allergology, Institute of Clinical Medicine, Vilnius University, Vilnius, Lithuania

**Keywords:** depression, mental health, conflict impact, PHQ-9 scale, geopolitical stress

## Abstract

**Background/Objectives:**

Amidst global conflict, mental health issues have surged, with a notable increase in Disability-Adjusted Life Years (DALYs) due to mental disorders from 1990 to 2019. In Lithuania, a high prevalence of depression contrasts with the global average, with a substantial healthcare allocation to mental health. The study aims to analyze depression rates across Lithuanian regions, employing the PHQ-9 questionnaire for accurate assessment, and addressing mental health needs through region-specific strategies.

**Methods:**

A cross-sectional study conducted in Lithuania employed an anonymous internet survey to assess the impact of conflict on mental health based on the PHQ-9 scale. Data analysis utilized statistical tools like Statistical Package for the Social Sciences (SPSS) and R studio. The study employed the Shepard operator for data interpolation, visualized in a detailed map of Lithuania illustrating regional depression levels. This approach is innovative as it utilizes advanced interpolation methods to create a highly accurate and detailed geographic representation of mental health data. The precision of this map allows for precise, location-targeted interventions for mental health prevention. This targeted approach is crucial in efficiently addressing mental health issues on a regional scale, ensuring that interventions are both effective and resource efficient.

**Results:**

Key findings included an increase in PHQ-9 depression scores from an average of 7.28 before the war to 9.00 two weeks after the invasion began, suggesting a possible association between the onset of conflict and depressive symptoms. The study revealed a moderate correlation (r = 0.443) between pre-war and post-war PHQ-9 scores. A linear regression model indicated factors affecting depression scores, including age and gender, and spatial mapping showed regional variations in depression, highlighting the western part of Lithuania and the north-eastern central region as areas with higher depression levels.

**Conclusions:**

The study shows increased depression symptoms after conflict, with diverse reactions across demographics and locations in Lithuania. Persistent severe symptoms and the varying effects of education and age on stress responses were observed. The research emphasizes the need for customized mental health strategies, particularly near geopolitical hotspots, to effectively address these challenges.

## 1 Introduction

### 1.1 Global war and mental health

Previously, it was emphasized that there is a significant need to raise awareness about the impact of armed conflicts on the mental health of refugees (1). It is known that the impact of war on mental health depends on a variety of factors, including some more common ones such as gender (2), but also some less apparent ones such as racial identity (3), which is why this subject demands a more detailed investigation. Data from Vietnam indicates that early life exposure to an armed conflict has a significant impact on mental health, affecting individuals well into their older years long after the exposure (4) and it is evident that the term “armed conflict” encompasses not only war but also exposure to gun violence in other situations unrelated to war (5). Targeted interventions based on various sociodemographic variables and locations (6) have proven effective in improving the mental health of residents, which is why the mapping method was employed in our study. However, the interventions required may be too complex, which is why international assistance in managing mental health in war-affected areas is essential (7).

The war greatly impacts society, especially education. This is extremely important considering that children are vulnerable in the context of war (8). A UNICEF report highlighted the severe effects of the Ukraine conflict on children’s education (9). In Ukraine, 5.3 million children face challenges accessing education, 3.6 million are affected by school closures, over 2,600 schools have been damaged, more than 400 are destroyed, and only about 25% of schools have provided full-time in-person learning since September 2022 (9). Globally, many individuals contribute to humanitarian efforts, assisting civilians and the injured (10). As of November 30, 2023, there are 78,677 personnel in 12 active UN peacekeeping missions from 121 countries, including experts, police, staff officers, troops, civilian personnel, and volunteers (10). Since 1948, there have been 71 peacekeeping operations. However, it has already been established that mental support should be extended not only to refugees but also to residents of other countries, particularly those who assist refugees (11).

While the statistics on armed conflicts paint a stark picture of their global prevalence and impact, it’s crucial to also turn our attention to the equally significant issue of mental health. Reflecting this, between 1990 and 2019, the global number of Disability-Adjusted Life Years (DALYs) due to mental disorders increased from 80.8 million to 125.3 million, and the proportion of global DALYs attributed to mental disorders rose from 3.1% to 4.9% (12). Out of this, war survivors, predominantly residing in low- or middle-income countries, bore a substantial burden of 3,105,387 and 4,083,950 DALYs associated with post-traumatic stress disorder (PTSD) and major depression (MD), respectively (13). Depression presents a significant societal challenge, and according to data from the Institute of Health Metrics and Evaluation, approximately 280 million people globally suffer from depression (14), constituting 3.8% of the world’s population. While definitive causal evidence directly linking military conflicts to increased disability-adjusted life years (DALYs) remains limited, a substantial body of research has established a robust association between psychological disorders and elevated DALY burdens. Given that military conflicts are consistently associated with heightened levels of psychological distress, including anxiety, depression, and post-traumatic stress disorder (PTSD), it is reasonable to hypothesize that such conflicts may indirectly contribute to an increase in DALYs by exacerbating the prevalence and severity of mental health conditions.

Refugees exhibit a high prevalence of mental disorders, with PTSD affecting 31%, major depression impacting 25%, and anxiety disorders present in 14% of this population (15). The same study notes that PTSD significantly impacts the younger generation. This data aligns with the prevalence of these disorders among residents living in war-torn conditions (16). The ongoing conflict in Ukraine, where affected populations are experiencing elevated rates of mental disorders (17), is likely to witness the same phenomenon. However, research indicates that disorders like PTSD may be associated with certain physical ailments (18). For instance, these populations observe temporomandibular disorder, a somatic condition characterized by pain upon muscle palpation.

Lithuania, recognized as one of the top 10 countries in the world with the highest rates of depression, exhibits a higher prevalence of this disorder compared to the global average, with 5.6% versus 4.4%, respectively, as of 2015. Depression is a mental health condition characterized by persistent feelings of sadness, a diminished interest in previously enjoyed activities, guilt or a reduced sense of self-esteem, disrupted sleeping or eating patterns, fatigue, and difficulty concentrating (19). Notably, Lithuania allocates 4.2% of its total health expenditure to mental health (20), which is significantly higher than the global median of 2.1% of government health expenditures typically observed, particularly in low- and middle-income countries (21). Despite having one of the highest ratios of psychiatrists per million residents in the EU, at 197 (20), Lithuania continues to face a crisis in the state of mental health. It requires significant enhancement, given that it ranks second globally in terms of suicide rates, with an annual statistic of crude rate 18.5 suicides per 100,000 residents (22).

The disparity in the diagnosis rates for mental disorders between Lithuania’s urban and rural areas is decreasing. However, there remains a significant challenge due to the uneven distribution of the primary mental health workforce, with rural areas facing a more acute shortage. Additionally, the incidence of diagnosed depressive disorders exhibits considerable variation among different Lithuanian municipalities (23). Establishing a detailed understanding of depression rates in various Lithuanian regions is vital, as it allows for the development of customized mental health prevention efforts specific to each area. This targeted approach is essential to effectively meet the unique mental health needs of different communities. A detailed analysis of depression rates across different regions can offer insights into how geographical factors and shifting geopolitical conditions may be associated with mental health outcomes. Furthermore, this objective forms the core of our study’s aim, underscoring our commitment to enhancing mental health care strategies through region-specific insights. One way to achieve this is by assessing the mental states of residents in different municipalities using the Patient Health Questionnaire-9 (PHQ-9) questionnaire, a tool validated for screening depression, which makes it an efficient and reliable method for gauging mental health across various communities (24). It is important to note, that the PHQ-9 scale has been employed in the past for evaluating the mental state of residents in situations characterized by stress and unpleasantness, such as during wildfires (25). Implementing the spatial interpolation method for mapping depression levels across Lithuania’s regions is essential. This innovative technique allows for a precise and detailed geographic portrayal of mental health statistics. Such a targeted approach is vital for effectively addressing mental health concerns at the regional level. It ensures that interventions are not only successful but also resource-efficient, offering a strategic advantage in pinpointing and responding to mental health needs in specific areas.

### 1.2 Theoretical framework

To interpret the observed associations between the onset of armed conflict and depressive symptoms in the Lithuanian population, this study draws upon several psychological and sociological models.

First, the stress-diathesis model suggests that individuals with underlying vulnerabilities (diathesis), such as previous mental health issues, socioeconomic stressors, or lack of social support, are more likely to exhibit depressive symptoms in response to external stressors like geopolitical conflict.

Second, Bronfenbrenner’s ecological systems theory emphasizes how individuals are embedded in multiple layers of influence—from family and community to national and global systems. The proximity of Lithuania to an active war zone may increase stress through media exposure, perceived threat, or community tension, thus affecting mental health.

Finally, the conservation of resources (COR) theory posits that stress occurs when individuals perceive a loss of resources—such as safety, stability, or emotional support. The onset of conflict in a neighboring country may threaten these resources, especially in border regions, explaining the geographic variability in depressive symptoms.

Integrating these frameworks enhances the explanatory power of our findings and helps interpret the demographic and regional patterns observed in the study.

## 2 Materials and methods

### 2.1 Ethics approval

The anonymous internet survey conducted in Lithuania did not require ethical approval according to local laws, as it did not qualify as a biomedical study. During the survey, the researchers were not privy to any personally identifiable information, ensuring that all participant responses were kept confidential and anonymous.

### 2.2 Study design

This research is a cross-sectional study that utilized cluster sampling. We created and administered one survey for this study using Google Forms.

The dissemination of the survey was conducted across 60 municipalities in Lithuania, with each municipality receiving an allocation of 50 questionnaires. It is important to clarify that two separate surveys were administered at distinct time points: one prior to the onset of the war and the second approximately one year later. Although the questionnaires were identical in content, they were completed by different participants in each wave, resulting in two independent cross-sectional samples. Data collection was conducted across 60 municipalities in Lithuania; however, response rates varied considerably, with some municipalities exhibiting very low participant turnout or no responses at all. This variation in regional representation should be taken into account when interpreting the spatial analysis and generalizability of the findings. Distribution channels included an online platform and a dedicated group composed exclusively of residents from the respective municipalities. This standardized distribution method was employed consistently across different periods of data collection.

In this study, it was conducted a statistical analysis to assess the power of the research design. The study comprised two groups with distinct sample sizes. The first group consisted of 1011 respondents. The questionnaire included items assessing participants’ current emotional state as well as their emotional state one week prior. It is important to note that, for respondents in the first data collection wave, the reference point of “one week ago” preceded the outbreak of the war, thereby providing a baseline measure uncontaminated by the immediate psychological impact of the conflict. The second group comprised 485 respondents. Our aim was to evaluate the statistical power of detecting a small effect size (*d* = 0.2) using a two-sided hypothesis test with an alpha level (α) set at 0.05.

Utilizing the T-test for independent samples, we calculated the statistical power of the study design. The power analysis revealed a statistical power of approximately 95.14%. This indicates a high likelihood of detecting a statistically significant effect if such an effect truly exists within the population.

The results suggest that the research design, with the specified sample sizes and effect size, provides substantial power to detect meaningful differences between the groups under investigation. These findings contribute to the robustness and reliability of the study outcomes and underscore the adequacy of the chosen sample sizes for achieving the research objectives.

### 2.3 Inclusion and exclusion criteria

This research included individuals living in Lithuania with internet access. Participants were not personally contacted or offered any incentives to participate in the study. This study did exclude 54 responses due to the respondent’s young age (under 18 years old).

### 2.4 Instruments and statistical analysis

We conducted an anonymous survey in March 2022, which included several components: 1) questions pertaining to demographic characteristics (age, gender, education, etc.), 2) the Patient Health Questionnaire-9 (PHQ-9) psychometric scale, and 3) inquiries related to the impact of war on their quality of life, work, and sense of national unity. This survey was carried out with 1,011 participants to assess their subjective mental and emotional state in relation to the onset of war and the pre-war period. Evaluations utilized the Patient Health Questionnaire-9 (PHQ-9), a widely employed tool for screening and assessing symptoms of depression. We initiated a new anonymous questionnaire survey in August 2023, targeting identical questions from the 2022 study.This questionnaire was refined – education categories were expanded to include primary and basic education, and questions regarding employment status (employed; unemployed; recipient of pension or capital income; student; others not part of the workforce such as volunteers, individuals not working under an employment contract; pregnant and postpartum women without an employment relationship; conscripts/military volunteers without an employment relationship; individuals without an employment relationship due to the constant care of a family member) were added, along with a question on job responsibilities for respondents who are employed. During this study, 485 respondents were surveyed. Upon collecting and processing the data, statistical analysis of the digitized questionnaires was performed using IBM SPSS Statistics 21 and R studio. The Shapiro-Wilk test was employed to determine that the distribution of the psychometric scale was not normal, thereby necessitating the use of non-parametric tests (Mann-Whitney U for two groups and Kruskal-Wallis or Friedman for multiple groups, respectively, along with Spearman’s correlation analysis). Finally, a linear regression model was developed, incorporating independent variables of national unity and work efficiency, with the depression scale serving as the dependent variable.

### 2.5 Integration with population data

In the pursuit of understanding regional variations in depression levels within Lithuania, a comprehensive survey collection was undertaken, followed by a spatial analysis. This approach aimed to spatially represent the survey data at a national scale without soliciting addresses directly from respondents.

The primary dataset comprised responses from a questionnaire capturing key demographic attributes of participants, such as age, gender, and educational attainment. Each respondent’s depression level was gauged based on the Patient Health Questionnaire (PHQ) score, and these scores were subsequently categorized into distinct levels of depression severity.

It is important to note that the PHQ-9 is a validated screening tool, not a diagnostic instrument. While it is widely used to assess the presence and severity of depressive symptoms, a clinical diagnosis of depression requires a structured clinical interview conducted by a qualified professional. In this study, PHQ-9 scores were interpreted using standard severity bands (e.g., minimal: 0–4, mild: 5–9, moderate: 10–14, moderately severe: 15–19, severe: 20–27), and findings are reported in terms of elevated depressive symptoms, rather than confirmed clinical depression. This distinction is critical to avoid overestimating the clinical implications of the results.

We also provided productivity and national unity questions. During the initial data preprocessing phase, participants’ ages were categorized into predefined brackets. These age brackets, along with other demographic information, were meticulously aligned with the Lithuanian National Statistical Department’s population survey data. It was noted that the national statistical department did not cover some mucipality of recidence in their 1x1 km grid data. For respondents from these areas, or those who did not specify their mucipality of recidence, points were generated based on the broader municipality region. However, a significant proportion, approximately 70% (345 out of 485 respondents), did provide specific mucipality of recidence information, enabling a more granular spatial representation for them.

The 1x1 km grid from the Lithuanian Statistical Department’s population census served as a foundational dataset (26). This grid, which detailed demographic distributions like age and gender, allowed for a seamless fusion of the survey data, thereby ensuring that the spatial representation mirrored the demographic dispersion of the population accurately.

To represent respondents spatially without violating privacy, an innovative approach to generating respondent coordinates was devised. Initially, the grid data was filtered based on each respondent’s age and gender. Subsequently, relevant boundaries were identified depending on the provided municipality and mucipality of recidence names. Within these boundaries, grids were selected randomly. The selection’s randomness was weighted primarily by the age demographic to ensure a representative selection. However, the methodology also boasts versatility by allowing for the incorporation of other demographic statistics as additional weights, if required. For each selected grid, a random point was generated to symbolize the respondent.

**Figure 1:**
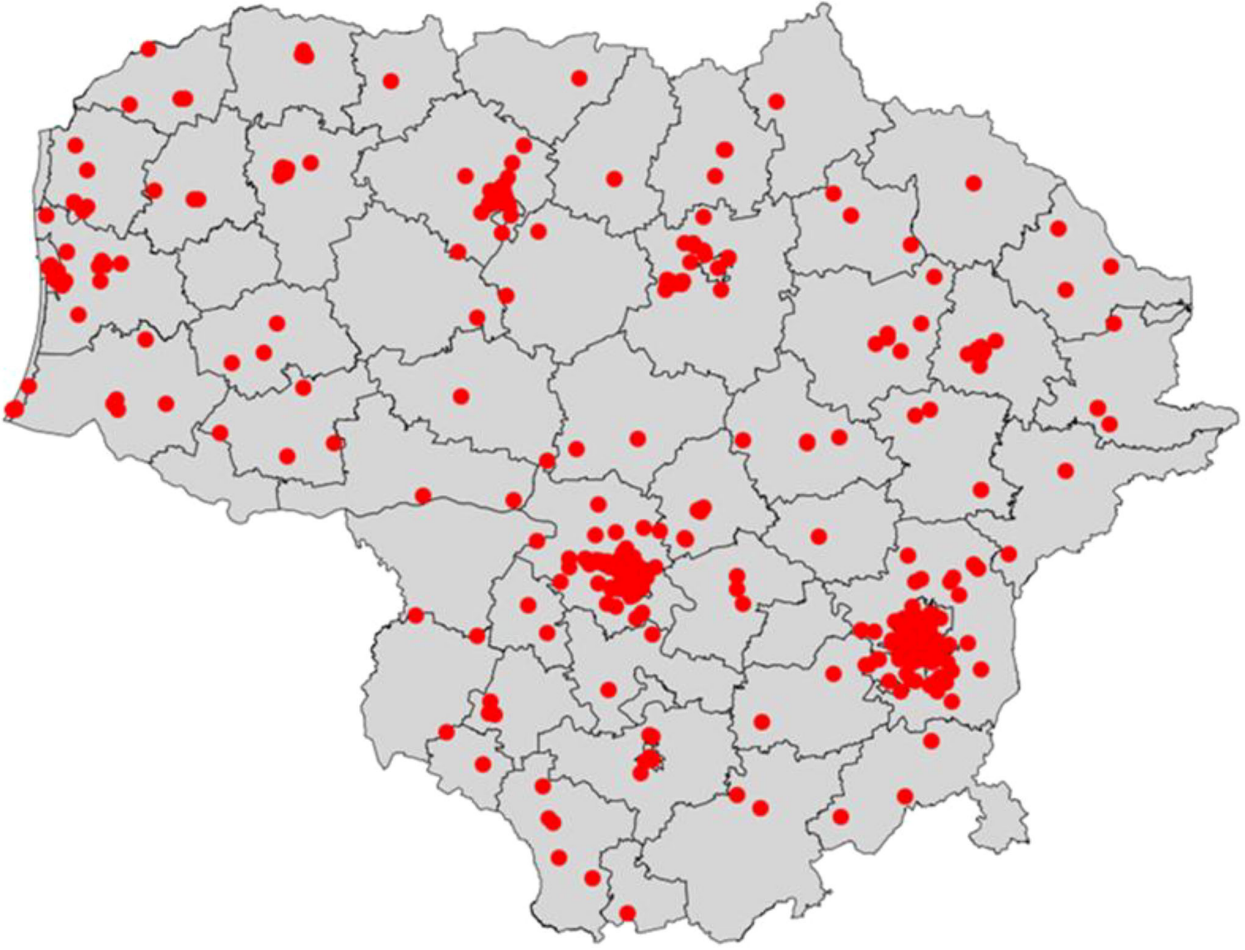
Spatial distribution of survey respondents across municipalities.

To validate and ensure the robustness of the generated coordinates, a spatial join with administrative boundaries was conducted. This step verified the alignment between the original and derived municipality names. Alongside this validation, a visual representation was crafted, wherein the selected points were plotted on a map, superimposed on the municipality grids (see **Figure 1**). This visualization provides a tangible grasp of the demographic and regional distribution of respondents, showcasing the granularity achieved by combining survey data with the population census grid.

Through this intricate fusion of survey data with the population census grid, the study offers a blueprint for future endeavors seeking to spatially represent sensitive data without compromising individual privacy.

Afterward, spatial interpolation techniques are being applied to generate a map of the country. The map is represented by 10,000 grids, which cover all of Lithuania and have information about depression levels, that is interpolated based on actual survey results.

### 2.6 Interpolation methods

Interpolation is a method for creating new data points within a set of known data points. The main interpolation methods can be categorized into three groups: deterministic, geostatistical, and those based on machine learning or deep learning. This discussion will not explore the latter. Deterministic interpolation involves generating surfaces from measured points, considering the degree of similarity.

### 2.7 Mapping

In this section, we present an interpolation algorithm that was specifically created for the map generation. This method has been implemented in the R programming language, and for the convenience of the reader, we have included the mathematical description.

### 2.8 The Shepard operator for scattered data interpolation

In this study, the Shepard interpolation method (Inverse Distance Weighting) was chosen due to its simplicity, computational efficiency, and previous success in similar public health applications. While geostatistical methods such as Kriging often provide more statistically robust estimations by modeling spatial autocorrelation, they also require stronger assumptions about the data distribution and spatial structure, which may not be fully met in this dataset. Additionally, Shepard’s method allowed for flexible application to a relatively sparse and unevenly distributed sample without complex variogram modeling. However, future studies could explore the use of Kriging or other advanced methods to compare accuracy and assess potential improvements in spatial resolution.

The Shepard operator, also known as Inverse Distance Weighted, is a well-established technique for interpolating scattered data, introduced by Donald Shepard (27). Among deterministic interpolation methods, it is widely utilized and recognized for its simplicity. The method involves calculating a weighted average of values at data points and is commonly applied in the interpolation of natural processes.

Consider dataset X=(x_1,x_2,…,x_K), where x_i∈d for 1≤i≤K and d≥1. The corresponding response values are given by Y=(y_1,y_2,…,y_K) where y_i∈R^p. Let us define a linear extrapolator as follows


y(x)=Y∧T u(x)

where


$$u\left(x\right)\;=\{\frac{w\left(x\right)}{1^{T}w\left(x\right)},\;if\;\;\left|x_{i}-x\right|\neq 0\;\forall i\;\;y_{i},\;\;if\;\left|x_{i}-x\right|=0$$

where 1 is a K – dimensional vector of ones, and the weights are determined by:


$$w\left(x\right)=\;\left(\frac{1}{{\left|x_{1}-x\right|}^{\delta }},\frac{1}{{\left|x_{2}-x\right|}^{\delta }},\ldots ,\frac{1}{{\left|x_{K}-x\right|}^{\delta }}\right)$$

with 0≤δ≤5. Note that the Shepard’s extrapolator satisfies the condition 1^T u(x)=1.

### 2.9 Map creation procedure

The following procedure outlines the steps for creating detailed maps by integrating both spatial and data-driven elements:

*1. Generate Grid:*

A structured grid system utilizing the shape file of the specific country of interest is created. This grid establishes a spatial framework crucial for subsequent stages of data interpolation.

*2. Input into Shepard’s Interpolator:*

The multivariate measurements are inserted into the previously generated grid.

*3. Interpolation to Grid Points:*

We apply Shepard’s operator to interpolate values across the entire grid. This step results in a comprehensive representation of geographic data.

## 3 Results

### 3.1 Data analysis

During the initial phase of the survey, which coincided with the onset of the war, a total of 1011 respondents participated, yielding a response rate of 33.7% (0.337). In contrast, a subsequent iteration of the survey conducted one year later garnered participation from 485 respondents, resulting in a reduced response rate of 16.2% (0.162).

The conducted study provides a comprehensive analysis of the respondents’ demographic composition and their subjective evaluations regarding certain issues. Respondents were categorized by gender, age, education, and region of residence, and their psychological indicators and subjective assessments were evaluated. The distribution of respondents by gender shows a predominance of the male population, constituting 63.2% of the total sample, compared to 36.8% of females. The average age of respondents is 27.6 years, with a standard deviation of 11.1 years, and the median age is 22 years. The data also indicate a broad age distribution among respondents, ranging from 18 to 78 years old. Regarding education, the majority of respondents, 46.8%, have secondary education, while 40.95% have higher university education. A smaller proportion of respondents possess higher college education 9.9% or lower than secondary education 2.4%. Regional characteristics revealed that the majority of respondents reside in Aukštaitija 68.4%. Psychological indicators, measured using the World Health Questionnaire-9, showed an increased average score during the war 9.00 compared to the pre-war period 7.3, with corresponding standard deviations of 5 and 4.7. Respondents’ subjective evaluations regarding the sense of national unity and work efficiency were also included in the analysis. The average evaluation of the sense of national unity was 8.04 with a standard deviation of 2, and the evaluation of work efficiency was 4.3 with a standard deviation of 2 (**Table 1**).

**Table 1:** displays the primary demographic characteristics of the 1011 respondents in the first survey.

Characteristics	n	%		
Gender (Male/Female)	372/639	36.8/63.2		
	Mean	Standard deviation	Median	Range
Age (year)	28.7	13.11	22	18-75
	n	%		
Education (Higher university degree)	414	40.95		
Education (Higher College)	100	9.89		
Education (Secondary)	473	46.76		
Education (Lower than secondary)	24	2.37		
Region of residence (Aukstaitija)	691	68.35		
Region of residence (Dzukija)	91	9.00		
Region of residence (Suvalkija)	89	8.80		
Region of residence (Zemaitija)	84	8.31		
Region of residence (Mazoji Lietuva)	56	5.54		
	Mean	Standard deviation	Median	Range
PHQ-9 (pre-invasion)	7.28	4.68	6	0-27
PHQ-9 (two weeks post-invasion)	9.00	4.96	8	0-27
Sense of belonging (1 - low; 10 - high)	8.04	2.02	8	01-10
Work efficiency rating^1^	4.26	2.04	4	01-10

1 - deterioration in work quality; 10 -improvement in work quality.

The study conducted allows for the identification of statistically significant relationships between variables. Notably, a moderate correlation is observed between the PHQ-9 scores pre-war and post-war onset, with a Spearman’s correlation coefficient of r = 0.443, p < 0.01, indicating a moderately positive relationship. This suggests that the respondents’ psychological state before and during the war changed consistently. Furthermore, a statistically significant negative relationship was identified between the pre-war PHQ-9 scores and the respondents’ age, r = -0.213, p < 0.001. This relationship indicates that older respondents had lower PHQ-9 scores before the war. A weak positive relationship is observed between the PHQ-9 scores after the war began and the respondents’ age, r = 0.081, p = 0.05. Changes in work efficiency and the sense of national unity are negatively correlated, however, this relationship is weak and statistically marginally significant, r = -0.066, p < 0.05. Other variables, such as age and the sense of national unity, change in work efficiency, show that these variables do not have statistically significant relationships. These results can be reviewed in **Table 2**.

**Table 2:** Correlation between demographic characteristics and scales assessing depression, national unity, and work efficiency.

	PHQ-9 (pre-invasion)	PHQ-9 (post-invasion)*	Sense of community	Work efficiency	Age
PHQ-9 (pre- invasion)	1.000				
PHQ-9 (post-invasion)^1^	0.443***	1.000			
Sense of community	0.015	0.062*	1.000		
Work efficiency	-0.030	-0.227	-0.067*	1.000	
Age	-0.213***	0.081**	-0.053	-0.034	1.000

***p < 0.001; **p < 0.01; *p < 0.05.

Two weeks after the start on the war.

Spearman’s correlation coefficients.

A linear regression model was developed wherein the dependent variable was scores obtained from the Patient Health Questionnaire-9 (PHQ-9), and the independent variables included gender, age, place of residence, change in work efficiency, and change in the sense of community. This regression model was constructed to assess and predict depression outcomes based on specific variables. The model’s coefficient of determination, R² = 0.329, indicates that 32.9% of the variation in depression scores can be explained by the model’s independent variables. Age: The study found that age is positively associated with PHQ-9 scores. Each additional year of age is associated with a 0.066-point increase on the PHQ-9 scale, indicating that older individuals tend to experience higher levels of depression. Impact of War on Work Efficiency: This factor is negatively associated with PHQ-9 scores. This suggests that individuals reporting reduced work efficiency in the context of the war were associated with lower depression scores. Pre-war PHQ-9 Score: As expected, the pre-war PHQ-9 score is strongly positively associated with the current PHQ-9 score. This indicates that the level of depression experienced in the past is a significant factor in predicting the current state of depression. Gender: The gender variable showed that men tend to experience a lower level of depression compared to women. This suggests that gender is an important factor influencing the level of depression. Education: The level of education also showed a significant relationship with the level of depression. Particularly, individuals with secondary education tend to have lower levels of depression compared to those with higher university and college education. Place of Residence: Residence in rural areas is associated with the PHQ-9 score, as individuals living in rural locations tend to experience higher levels of depression (**Table 3**).

**Table 3:** Linear regression model where the scale of depression symptoms is the dependent variable, and independent variables include age, place of residence, education level, assessments of the impact of war on work efficiency, and national unity.

Independent variable	Reg. coefficient β	Standard error	t value	p value
Constanta	6.16	0.868	7.097	<0.001***
Age	0.066	0.014	4.694	<0.001***
Gender [T.Male]	-1.878	0.276	-6.794	<0.001***
Education [T.Higher College]	0.133	0.461	0.288	0.773
Education [T.Secondary]	-0.759	0.319	-2.377	0.018*
Education [T.Lower than secondary]	0.319	0.896	0.356	0.722
Place of Residence [T.City]	-0.562	0.29	-1.939	0.053.
Place of Residence [T.Town]	-0.731	0.548	-1.334	0.183
Place of Residence [T.Village]	-0.046	0.791	-0.058	0.954
Place of Residence [T.Rural Area]	4.601	1.698	2.71	0.007**
Region [Dzukija]	0.909	0.461	1.972	0.049*
Region [T.Minor Lithuania]	0.86	0.581	1.482	0.139
Region [Suvalkija]	0.271	0.472	0.574	0.566
Region [Zemaitija]	0.531	0.498	1.067	0.286
Impact of War on Work Efficiency	-0.341	0.065	-5.278	<0.001***
Impact of War on National Unity	-0.008	0.066	-0.124	0.901
Pre-invasion PHQ-9	0.492	0.029	17.124	<0.001***

In the second survey, 485 participants were involved, with their primary demographic characteristics presented in **Table 4**. This study conducted a quantitative assessment to examine the demographic characteristics and psychosocial indicators of the respondents. The study included 485 participants, of whom 162 (33.4%) were males, and 323 (66.6%) were females. The average age of the respondents was 28.7 years (SD = 13.1), with an age fluctuation range from 18 to 75 years. The median age was 22 years. The education level was divided into six categories: higher university, higher college, secondary, primary, elementary, and no education. The majority of respondents (n = 254; 52.4%) had secondary education, and 169 respondents (34.9%) had higher university education. Employment status was categorized into five groups: employed individuals, students, pension or capital income receivers, unemployed individuals, and others not included in the workforce. The majority of respondents were students (n = 250; 51.6%) or employed individuals (n = 199; 41%). The PHQ-9 index, measuring the severity of depression a year after the start of the war, had an average score of 7.53 (SD = 5.9), with a range of scores from 0 to 27. The sense of national unity was assessed on a ten-point scale, where 1 indicated a low feeling, and 10 indicated a high one. The average rating was 7.00 (SD = 2.6). The assessment of work efficiency was measured on a ten-point scale, where 1 indicated that the quality of work had deteriorated, and 10 indicated it had improved. The average rating was 4.76 (SD = 1.6). These data provide detailed information about the demographic composition and psychosocial indicators of the participants in the study, allowing for a better understanding of the social and psychological consequences of the war (**Table 4**).

**Table 4:** Demographic, depression, work efficiency, and unity sentiment evaluation characteristics (one year after the war’s onset).

Characteristics	n	%		
Gender (Male/Fermale)	162/323	33.4/66.6		
	Mean	Standard deviation	Median	Interval
Age (year)	28.6866	13.11302	22	18-75
	n	%		
Education (Higher university degree)	169	34.85		
Education (Higher College)	44	9.07		
Education (Secondary)	254	52.37		
Education (Elementary)	16	3.30		
Education (Primary)	1	0.21		
Education (Absent)^1^	1	0.21		
Employment (Employed)	199	41.03		
Employment (Student)	250	51.55		
Employment (Capital income)^2^	17	3.51		
Employment (Unemployed)	10	2.06		
Employment (Other)^3^	9	1.86		
	Mean	Standard deviation	Median	Interval
PHQ-9 (one-year post-invasion)	7.527	5.928	6	0-27
Sense of belonging (1 - low; 10 - high)	6.997	2.570	8	01-10
Work efficiency rating^4^	4.762	1.605	5	01-10

1. Without any education or training

2. Pension or capital income beneficiary

3. Does not depend on labor

4. 1 - deterioration in work quality; 10 - improvement in work quality

Spearman’s correlation analysis was conducted to explore the relationships between depression symptoms measured by the PHQ-9 scale one year after the onset of the war, the sense of national unity, changes in work efficiency, and respondents’ age. The PHQ-9 scale, which ranges from 0 to 27, was utilized to assess the severity of depression symptoms, where higher values indicate greater severity of depression. Assessments of the sense of national unity and changes in work efficiency were conducted using a 10-point scale, where lower values indicate a negative effect and higher values indicate a positive effect. The results revealed that there was a weak negative correlation between PHQ-9 scores and changes in work efficiency (r = -0.149, p < 0.001), indicating that higher symptoms of depression are associated with lower work efficiency. A weak negative correlation was also found between PHQ-9 scores and age (r = -0.296, p < 0.001), showing that younger respondents experience higher symptoms of depression. The sense of national unity and changes in work efficiency also had a negative correlation (r = -0.122, p < 0.01), depicting that a stronger sense of national unity is associated with lower work efficiency. The sense of national unity and age also had a weak negative relationship (r = -0.161, p < 0.001), as the study demonstrated that younger respondents feel a stronger sense of national unity (**Table 5**).

**Table 5:** Camong demographic variables and scales assessing depression, national unity, and work efficiency.

	PHQ-9 (post-invasion)	Sense of community	Work efficiency rating	Age
PHQ-9 (post-invasion)^1^	1.000			
Sense of community	0.063	1.000		
Work efficiency rating	-0.149***	-0.122**	1.000	
Age	-0.296***	-0.161***	-0.033	1.000

***p <0.001; **p<0.01; *p<0.05.

1. One year after the war has started.

The model (with a determination coefficient of R^2 = 0.1119) revealed several important factors. For instance, compared to women, men had an average PHQ-9 score that was 2.11 points lower, indicating that men experience less severe symptoms of depression than women. Furthermore, with each passing year, the PHQ-9 score decreased by 0.10 points, suggesting that older individuals might experience slightly less severe symptoms of depression. Regarding education, respondents with secondary education, compared to those with primary education, had an average PHQ-9 score that was 2.5 points higher, indicating that the level of education might influence the severity of depression symptoms. Finally, for each point increase in the variable assessing how the war in Ukraine affects work efficiency, the PHQ-9 score decreased by 0.58 points, reflecting that the impact of the war in Ukraine on work efficiency may be associated with the severity of depression symptoms. Although the model explains only about 11.2% of the variability in the PHQ-9 scores, it provides important preliminary information about the causal changes in the severity of depression symptoms and lays the groundwork for further research in this area (**Table 6**). The regression models used in this study explained a relatively small proportion of the variance in depression scores (e.g., R² = 0.1119), suggesting that additional unmeasured factors likely influence depressive symptoms. This limitation should be acknowledged when interpreting the findings. To assess multicollinearity among predictors, we calculated variance inflation factors (VIFs), and all values were below the commonly accepted threshold of 5, indicating no substantial multicollinearity. Residual plots were visually inspected and did not reveal major violations of homoscedasticity or linearity assumptions. However, the low R² values suggest the need for caution in interpreting the explanatory strength of the models. Future studies could improve model fit by including more psychosocial variables or using hierarchical/multilevel modeling approaches.

**Table 6:** Linear regression model with depression symptoms scale as the dependent variable and independent variables including age, place of residence, education, impact of war on work efficiency, and national unity ratings.

Independent variable	Reg. coefficient β	Standard error	t value	p value
Constanta	13.036	1.847	7.057	<0.001***
Gender [T.Male]	-2.110	0.555	-3.801	<0.001***
Age	-0.101	0.033	-3.007	0.002**
Education [T.Higher education]	0.513	0.959	0.535	0.592
Education [T.No education]	6.500	5.934	1.095	0.273
Education [T.Elementary education]	1.854	1.604	1.155	0.248
Education [T.Primary education]	-3.164	5.684	-0.557	0.578
Education [T.Secondary education]	2.502	0.895	2.795	0.005**
Employment [T.Other]	0.690	1.926	0.359	0.720
Employment [T.Not employed]	3.083	1.861	1.657	0.098
Employment [T.Capital income]^1^	2.745	1.689	1.625	0.104
Employment [T.Student]	-1.309	0.919	-1.425	0.154
Sense of community	-0.005	0.105	-0.056	0.955
Work efficiency rating	-0.581	0.161	-3.602	<0.001***

Recipient of pension or capital income.

**Table 7** depicts the percentage distribution of depression symptoms across three different periods: before the war, at the onset of the war, and one year after the war’s start. Within each depression symptom group, we observe how the percentage of symptoms changes over time. Key findings include: Minimal depression symptoms (0–4 points) constituted the majority of all cases before the war. However, with the onset of the war, the percentage of this group decreased, and one year after the war began, it increased again but did not exceed the initial level before the war. Mild depression symptoms (5–9 points) also experienced a decrease in percentage one year after the war’s start compared to previous periods. On the other hand, moderate depression symptoms (10–14 points) increased in percentage at the war’s onset, and one year after the war began, this group decreased but was still higher than before the war. Finally, severe depression symptoms (15–19 points) increased in percentage at the onset of the war, and one year after the war began, this group decreased but was still higher than before the war.

**Table 7:** Distribution of depression symptoms across three different periods: before the war, upon the start of the war, and one year after the start of the war.

	Before the war (total respondents 1013)		At the onset of the war (total respondents 1013)		One year post-invasion (total respondents 485)	
Depression symptoms	Frequency	Percentage	Frequency	Percentage	Frequency	Percentage
Minimal	303	29.9	184	18.4	180	37.1%
Mild	463	45.7	408	40.3	167	34.4%
Moderate	169	16.7	283	27.9	77	15.9%
Severe	55	5.4	103	10.2	33	6.8%
Very Severe	23	2.3	33	3.3	28	5.8%

Data analysis revealed that the median PHQ-9 scores, measuring levels of depression, differed between male and female groups before the war period (p<0.01). This trend was repeated during the war and one year after the start of the war (p<0.01). It was also observed that individuals before and after the war with higher university and college education had lower PHQ-9 scores compared to respondents with secondary and below-secondary education. During the war, the PHQ-9 median was observed to be one point higher for individuals with higher university and college education compared to those with secondary and lower education (**Table 8**).

**Table 8:** PHQ-9 scale scores by gender and education across different periods using the Mann–Whitney U test (median).

	PHQ-9		
	Before the war	During the war	One year post-invasion
Male	6	7	7
Female	7	10	6
p-value	0<0.01		
Higher University Education	6	9	5
Higher College Education	6	8.5	4
Secondary Education	7	8	8
Below Secondary Education	8	8	7
p-value	p<0.01		

The results of the Friedman test indicate statistically significant differences in PHQ-9 depression scale scores across three study periods: before the war, during the war, and one year after the war began (p<0.001). Median scores revealed that the level of depression was lowest before the war (median = 6), increased during the war (median = 10), and then decreased again one year after the war started (median = 6). These findings demonstrate that the events of the war had a negative impact on the psychological state of the participants, and the level of depression returned to its initial level one year after the onset of the war (**Table 9**). These conclusions highlight the need for psychological support and assistance for those experiencing stress caused by the war, indicating that the impact of war can be temporary, but its consequences may be long-lasting. This is an important message for policymakers, healthcare professionals, and the public to understand and address the psychological challenges posed by war.

**Table 9:** Assessment of the PHQ-9 scale over different periods using the friedman test.

PHQ-9			p-value
Before the war	During the war	One year post-invasion	
6	10	6	p<0.001

### 3.2 Mapping

**Figure 2:**
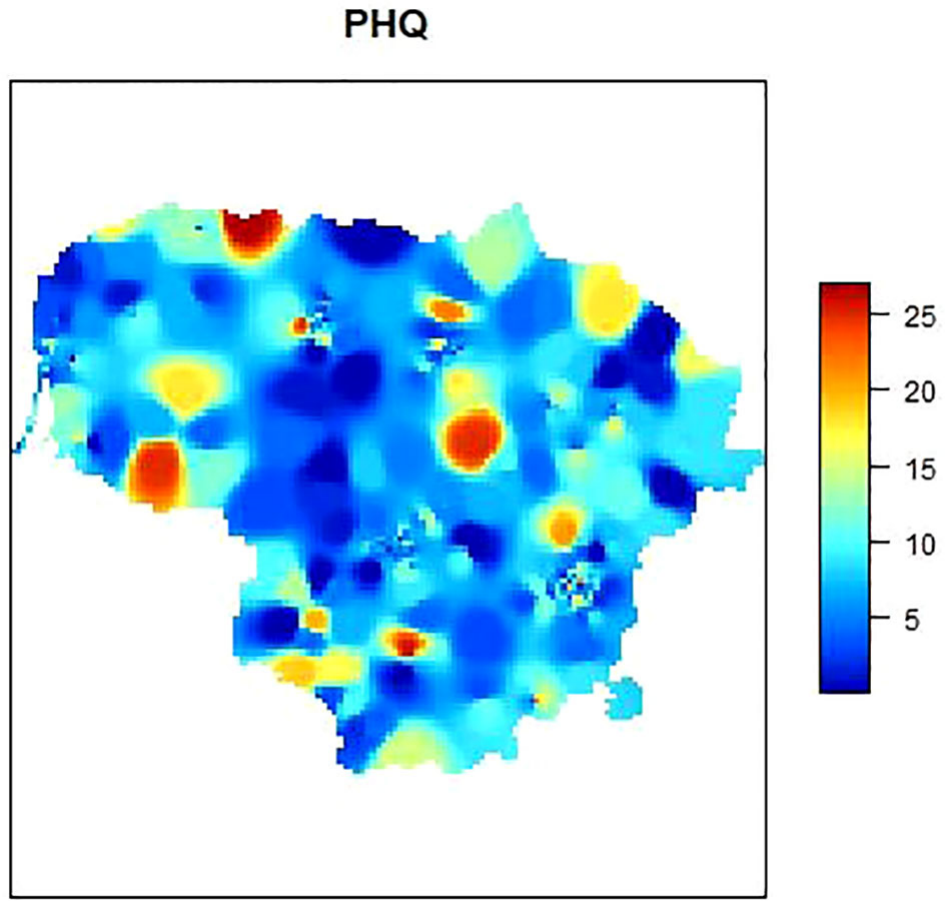
Geographic distribution of depression severity in Lithuania after the Russian invasion of Ukraine.

The image appears to be a heat map representing the Patient Health Questionnaire (PHQ) scores across a specific geographic area, which, based on the shape, could be a stylized representation of a country or region. **Figure 2** illustrates a heat map of Lithuania, delineating the severity of depression among regions one year after the Russian incursion into Ukraine. This cartographic representation employs a color gradient to signify the prevalence of depression, with cooler hues—represented by blue—indicating regions with lower depression levels, and warmer hues—denoted by red—highlighting areas with heightened depression levels, suggesting a concentration of individuals with severe depressive symptoms.

The map distinctly indicates that the western part of Lithuania, which shares a border with the Russian enclave of Kaliningrad, exhibits higher depression levels. The presence of such severe depressive symptoms may be associated with geopolitical stressors related to proximity to an aggressive neighbor. Furthermore, an intense concentration of severe depressive symptoms is evident in the north-eastern central region, as well as in the north-western part. The latter may suggest a regional pattern that warrants further investigation.

This innovative approach to data visualization transcends traditional methodologies applied to depression scales and provides a novel lens to comprehend the distribution of mental health challenges. The implications of such a visualization are multifaceted. Firstly, it enables targeted interventions by allowing policymakers and mental health professionals to identify regions with acute needs for psychological support. Secondly, it facilitates the optimization of resource allocation, potentially reducing healthcare expenditures by directing efforts and funds to the most affected locales. Lastly, the heat map may serve as an indicator of the infrastructure deficits within the mental health sector, particularly highlighting the potential scarcity of mental health professionals, such as psychologists and psychiatrists, in the regions that are most afflicted.

The current analysis underscores the need for a strategic approach to mental health services in Lithuania, one that is responsive to the geographical disparities revealed through this assessment. By adopting such geographically nuanced strategies, there is potential not only to enhance the well-being of individuals but also to contribute to the overall resilience of communities in the face of ongoing geopolitical tensions.

## 4 Discussion

### 4.1 Description of data and analysis with international data

The data reveal a pronounced increase in mean PHQ-9 scores from 7.28 pre-conflict to 9.00 immediately post-conflict, indicating significant distress. Notably, this heightened level subsides to 7.52 within a year, suggesting adaptive coping responses in the population. In a country experiencing war, the scenario is notably different, as the demand for mental health support remains consistently high throughout the conflict (28). This suggests that mental adaptation mechanisms might be less effective under the prolonged stress and uncertainties of war conditions.

While a group of respondents with minimal depression symptoms initially dominated, it decreased post-conflict onset, only partially recovering a year later. ‘Moderate’ and ‘Severe’ symptom groups saw an increase at the war’s start, remaining elevated after a year, despite some decrease. This dynamic may reflect an immediate psychological response to the onset of the war, which was associated with an increase in more severe depression symptoms. However, in Lithuania, the proportion of respondents exhibiting severe depression symptoms was significantly lower, nearly four times lower than in the war-affected country (29). This significant contrast underscores the differing levels of mental health impact between a nation adjacent to a conflict zone and one at the center of the conflict.

Based on the data from the Czech Republic’s student cohort, they reported a sustained higher mean score of 8.66 at the one-year mark, highlighting differing psychological impacts and coping mechanisms in varied demographic and geopolitical contexts (30). It is important to note, however, that the initial PHQ-9 score of Czech students immediately following the onset of the war remains unknown. This gap in data presents a limitation in directly comparing the immediate mental health impacts of the conflict between the two populations.

We found a significant negative correlation between pre-war PHQ-9 scores and respondents’ age, suggesting that older individuals had lower depression scores before the war. This observed trend of decreasing depression with age aligns with typical patterns identified in previous studies (31). However, it’s noteworthy to mention that a study from Korea presents an alternative perspective, suggesting a U-shaped dependency of PHQ-9 scores on age (32). Interestingly, a weak positive correlation was observed between “post-war” PHQ-9 scores and age, with each additional year of age correlating to a 0.066-point increase in PHQ-9 score. This trend could suggest that older individuals generally exhibit greater resilience to stress under normal circumstances, but this resilience may not extend to periods of significant geopolitical changes and the looming threat of war.

An interesting negative correlation emerged between the impact of war on work efficiency and PHQ-9 scores, indicating that those who experienced reduced work efficiency due to war tend to report lower depression scores. The negative correlation between decreased work efficiency due to war and lower depression scores could be attributed to a shift in priorities and increased resilience, along with enhanced community support and adaptive coping strategies during crises. A year later, the scenario shifted, with the correlation analysis showing that higher depression scores were associated with decreased work efficiency. In this scenario, our data align with findings from other studies which suggest that depression is linked to a decrease in work efficiency (33).

The initial survey conducted right after the war started recorded a higher sense of national unity (8.04) and lower work efficiency (4.26). A year later, the unity scores moderately decreased to 7.00, while work efficiency almost stayed at the same level showing a marginal increase to 4.76. This observation aligns with existing research showing that individuals who have experienced depression are more likely to face unemployment (34).

A year after the conflict in Ukraine, the regression analysis revealed that Lithuanian men, on average, scored 2.11 points lower than women on the PHQ-9, indicating less severe depression symptoms.This aligns with findings from other studies but presents a paradox given the higher suicide rates typically observed among males (35).

We observed notable variations in PHQ-9 scores based on educational attainment before, during, and after the war. Before and a year after the war started, individuals with higher university and college education generally had lower PHQ-9 scores than those with secondary or below-secondary education. This pattern was earlier established not only for depression in regular situations but also for postpartum depression (36) and depression occurring after a stroke (37). However, right at the beginning of the armed conflict, there was an interesting shift: the median PHQ-9 score for individuals with higher education rose by one point compared to those with lower educational levels. The different ways that educational levels influence individuals’ coping mechanisms and their perception of stressors could explain this trend.Higher education could be associated with better access to resources, information, and coping strategies that reduce stress and depression in normal times. However, during acute geopolitical crises, these advantages might be less protective, leading to a relative increase in stress and depression among the more educated population.

The sample accurately reflected the regional demographics of Lithuania. To achieve a more detailed representation at the municipal level, spatial interpolation was employed for data visualization. Our study is distinguished by its utilization of spatial interpolation to accurately visualize mental health data on a map, depicting the mental state of residents. This method allows for a precise and clear representation of the data across different regions, highlighting its uniqueness in the field of mental health research. This method had previously been applied in mapping health attitudes related to COVID-19 (38). According to studies conducted earlier, this kind of studies “can be used to predict the survey data for all non-enumerated areas” (39). Western Lithuania, bordering the Russian enclave of Kaliningrad, displays heightened depression levels, possibly due to geopolitical stressors from this proximity. Additionally, significant depressive symptoms are concentrated in the north-eastern central and north-western parts, suggesting regional patterns that merit further exploration. The direct geopolitical stressors associated with this proximity may be linked to the heightened depression levels observed in western Lithuania, which borders the Russian enclave of Kaliningrad. The close border with a region that has experienced significant geopolitical tension likely exacerbates mental health concerns among the local population. Furthermore, the concentration of depressive symptoms in the north-eastern central and north-western parts of Lithuania suggests regional patterns that could be influenced by a combination of factors. These might include economic conditions, historical and cultural contexts, or variations in access to mental health resources. The presence of these symptoms in specific regions implies that certain areas may be more vulnerable to mental health challenges due to their unique socio-economic and historical circumstances.

### 4.2 Potential suggestions for policymakers

Based on the study’s findings and the unique aspects identified, several suggestions for policymakers can be made. First, there is a need for a clear regional focus, as significant variations in depression rates across different areas highlight the necessity for targeted mental health strategies. Support systems should also be developed specifically for high-risk groups such as younger individuals or those with less education. These could include counseling services, mental health education in schools, and community-based programs. Another essential recommendation is the integration of mental health assessments and treatments into primary healthcare systems to ensure early detection and intervention.

Policymakers are encouraged to fund further longitudinal studies to monitor the enduring mental health effects of geopolitical stress and military conflicts on populations. This understanding would aid in assessing the long-term impact and effectiveness of interventions.

It is crucial to develop policies that ensure adequate training and support for mental health professionals working in high-stress and conflict-impacted regions. Improving technology, such as telepsychiatry, could provide mental health services in remote areas, ensuring accessible care for all residents. Lastly, implementing systems for continuous monitoring and evaluation of mental health strategies is essential to ensure their effectiveness and to make necessary adjustments. These comprehensive steps aim to improve the overall mental health infrastructure in response to military conflicts and their effects on neighboring regions.

### 4.3 Potential confounding by COVID-19

It is important to acknowledge that the first data collection period (March 2022) overlapped with the tail end of COVID-19 restrictions in Lithuania. The pandemic itself, along with its social and economic consequences—such as isolation, uncertainty, and changes in employment or education—has been shown to significantly impact mental health. Therefore, the observed depressive symptoms in the initial phase of this study may reflect not only reactions to the geopolitical conflict but also residual effects of the COVID-19 pandemic. Although the current study did not control for this confounding factor, future research should consider COVID-19-related stressors when interpreting depression trends during overlapping crises.

## 5 Limitations of the study

### 5.1 Geographical and cultural limitations

The findings are specific to Lithuania and might not be generalizable to other regions or cultures. Differences in healthcare systems, cultural attitudes towards mental health, and exposure to conflict might influence depressive symptoms differently across populations.

### 5.2 Sampling methodology

The study utilizes convenience sampling, which may not represent the entire population. This method might lead to selection bias as it does not guarantee that every individual in the population has an equal chance of participating. The use of an internet survey might limit participation to individuals with internet access, potentially excluding certain demographic groups who might be more or less susceptible to depression.

### 5.3 Cross-sectional design

As a cross-sectional study, it captures data at a single point in time or over a short period. This design limits the ability to determine causality between the observed effects and the military conflict.

### 5.4 Reliance on self-reported data

The study is based on responses to the PHQ-9 questionnaire, which are self-reported and may be subject to bias such as exaggeration, underreporting, or participants’ mood at the time of completing the survey.

### 5.5 Lack of detailed demographic data

While the study includes demographic data, more detailed information might be needed to better understand how different groups (e.g., based on socioeconomic status, ethnicity, or previous mental health history) are affected.

### 5.6 Sampling bias and representativeness

One of the key limitations of this study is its reliance on online convenience sampling, which may have introduced selection bias. This sampling method tends to disproportionately represent younger, more educated, and urban populations—particularly those with greater internet access and engagement in digital platforms. As a result, certain groups, such as older adults, individuals from rural areas, or those with lower levels of education, may be underrepresented. This limitation affects the generalizability of the findings to the broader Lithuanian population. Future research could improve representativeness by employing probabilistic sampling or incorporating post-stratification weights based on demographic data (e.g., age, gender, region, education) to adjust for discrepancies between the sample and national population structure.

## 6 Conclusions

The data indicates that depression symptoms increased in parallel with the onset of conflict, with a notable difference in adaptation and coping mechanisms across different demographics and geographical locations. The data indicated a slightly higher depression score in Lithuania compared to the figures from the Czech Republic. Within a year, we observed some resilience and recovery, but severe symptoms persist, especially in certain areas of Lithuania. Educational attainment and age show complex interactions with depression levels during and after the conflict, suggesting that factors like higher education and age may influence individual responses to stress differently. Additionally, regional disparities in Lithuania, particularly near geopolitical hotspots, highlight the impact of local factors on mental health.

The need for customized interventions in mental health care varies with the geopolitical environment. During peaceful times, emphasis should be placed on preventive measures targeting younger, less educated populations. In contrast, during conflict, the focus should shift to supporting older, more educated individuals. The study highlights the importance of geographically specific interventions, particularly in regions near Russia and in certain Lithuanian areas.

The proposed method, integrating spatial interpolation, offers enhanced accuracy and effectiveness in mental health care strategies. We tailor this innovative method, which employs spatial interpolation to precisely visualize mental health data on a map, to meet the diverse needs of various population segments, taking into account their geographic locations and exposure to geopolitical events. This approach not only improves the precision of mental health monitoring but also directs targeted mental health interventions, which have proven effective in previous studies that were mentioned earlier. The detailed maps allow for the strategic implementation of specific interventions in the most needy regions. Hospitals should bolster their mental health training for staff to enhance identification and treatment efforts in regions with high incidences of mental health concerns as indicated by the map’s data. This specialized focus is critical for societal well-being, where increased educational initiatives can foster greater understanding and support for mental health challenges. Government bodies are encouraged to direct funds and policy development toward mental health facilities in critical need areas, taking guidance from the spatial analysis. Additionally, NGOs play a vital role in filling service gaps, advocating for mental health awareness, and working in tandem with local communities, particularly where funding and resources are scarce. These collaborative efforts are essential for creating a supportive network addressing mental health needs on both local and broader scales.

## Data Availability

The raw data supporting the conclusions of this article will be made available by the authors, without undue reservation.
